# An Assessment of Airborne Bacteria and Fungi in the Female Dormitory Environment: Level, Impact Factors and Dose Rate

**DOI:** 10.3390/ijerph19116642

**Published:** 2022-05-29

**Authors:** Yanju Li, Xinyu Wang, Guoqing Cao, Yu Wang, Qingqing Miao, Jinlu He

**Affiliations:** 1 School of Energy and Safety Engineering, Tianjin Chengjian University, Tianjin 300384, China; cjjh20123@126.com (X.W.); q15936987091@163.com (Q.M.); hl191199@163.com (J.H.); 2 Institute of Building Environment and Energy, China Academy of Building Research, Beijing 100013, China; caoguoqing@chinaibee.com

**Keywords:** indoor environment, bacteria, fungi, impact factors, dose rate

## Abstract

In this study, the levels of airborne bacteria and fungi were tested in a female dormitory room; the effects of heating, relative humidity and number of occupants on indoor microorganisms were analyzed and the dose rate of exposure to microbes was assessed. The bacterial and fungal concentrations in the room ranged from 100 to several thousand CFU/m^3^, and the highest counts were observed in the morning (930 ± 1681 CFU/m^3^). *Staphylococcus* spp. and *Micrococcus* spp. were found in the dormitory. When the heating was on, the total bacterial and fungal counts were lower than when there was no heating. Moreover, statistically significant differences were observed for bacterial concentrations during the morning periods between the times when there was no heating and the times when there was heating. The number of occupants had an obvious positive effect on the total bacterial counts. Moreover, RH had no correlation with the airborne fungi in the dormitory, statistically. Furthermore, the highest dose rate from exposure to bacteria and fungi was observed during sleeping hours. The dose rate from exposure to airborne microorganisms in the dormitory was associated with the activity level in the room. These results helped to elucidate the threat of bioaerosols to the health of female occupants and provide guidance for protective measures.

## 1. Introduction

Modern humans spend more than 90% of their time in indoor environments [1,2]. In addition to particulate and chemical pollutants, airborne biological contaminants impact the indoor environment and pose a risk to human health [3,4]. As people spend significant amounts of time indoors, an understanding of the concentrations of airborne biological pollutants in these environments is urgently and needed.

To date, several studies have addressed both bacterial and fungal pollution in the air inside dwellings, and have reported concentrations of bacteria and fungi of approximately 10^2^–10^4^ colony-forming units (CFU) m^−3^ [5,6,7,8]. Environment-related factors such as temperature, relative humidity (RH), ventilation, season, outdoor climate and number of occupants can affect the density and distribution of airborne microbes detected indoors [9,10,11,12,13,14,15,16,17]. However, the fungi found in homes are primarily influenced by various characteristics of the homes [18]. In addition, human activities and gender have an observable influence on airborne bacteria and fungi [19,20]. Heating and cooling also have an impact on the indoor airborne microbes [21], but few studies have addressed the effects of indoor heating, which is widely used in the northern China, on biological pollutants.

In the field of aerobiology, airborne microbial pollutants have been found to have negative effects on health and indoor air quality [22,23]. In field studies, the species of *Micrococcus*, *Staphylococcus*, *Cladosporium*, *Penicillium* and *Aspergillus* were detected in indoor environments [24,25]. Moreover, microbial hazards are not only closely related to concentration and species, but also to contact time (“exposure”). Exposure to microorganisms has become a worldwide concern in environmental science and public health research. The majority of the previous work on dose rate has focused on chemical and particulate matter [26,27,28]. Assessment of microbe exposure is more challenging because, unlike chemical pollutants, microbes are not covered by general air quality standards and have thousands of species. Few studies focused on exposure to microorganisms in indoor environments.

In field studies, indoor airborne bacteria and fungi were detected in air and dust, and widely existed in the dormitory environment [6,19,29,30,31]. The richness and abundance of indoor bacteria were positively associated with infections [32] and were affected by the level of occupancy in the dormitory. Several million young women are accepted into universities every year in China. Many of them live in shared dormitory rooms, where they spend large portions of their time with potential exposure to environmental pollutants. Previous field studies have reported that the peak concentration of the microbes appeared in winter, and no differences were observed in total bacterial counts between the winter and summer [33,34]. Crowded female dormitories have more bacteria than male dormitories [35] and people spend more time in them during cold seasons. Microbial pollution in female dormitories has not been sufficiently addressed, particularly in regard to the effect of heating and the parameters of occupant number and dose rate. Therefore, the main objectives of this study were to assess the dose rate of bacteria and fungi in female dormitories and to investigate the effects of heating, environmental factors and number of occupants on the levels of bacteria and fungi indoors.

## 2. Materials and Methods

### 2.1. Sampling Positions

**Figure 1:**
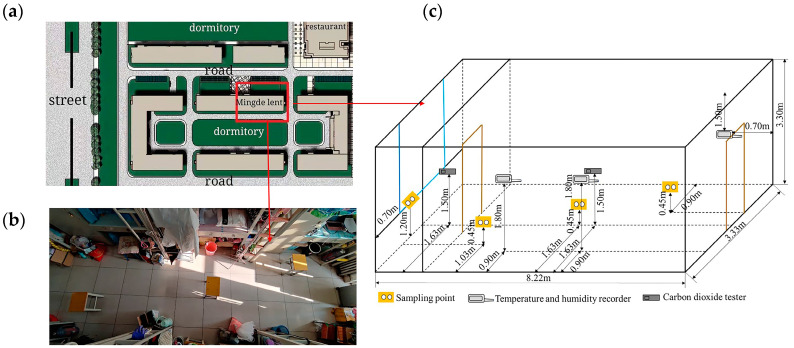
Layout of the tested dormitory and the indoor sampling positions: (**a**) building location; (**b**) indoor view of room; (**c**) sampling points.

The experiments were conducted in a female dormitory with natural ventilation at a university in Xiqing District of Tianjin, China (117.09605° E, 39.09703° N). The selected room (8.2 m × 3.3 m × 3.3 m) was located on the second floor of the dormitory, which is surrounded by trees and far away from the interchange traffic. The layout of the tested room, occupied by six females and six sets of two-level furniture, is shown in Figure 1a,b. The measured points are shown in Figure 1c. Temperature and RH were sampled at a height of 1.8 m above the floor, on the three handrails of the bed [36]. Based on the national standard [36], the indoor CO_2_ was tested in the center of the room at one sampling point. The outdoor CO_2_ was tested outside the window. Three indoor sampling points for bacteria and fungi were located at a height of 0.9 m, under the sampling positions for temperature. The sampling points for microbes outside of the building were 1.2 m from the floor and 0.7 m away from the wall.

### 2.2. Sampling Time and Instruments

The sampling period was from 12 October 2020 to 26 November 2020 (the room was heated from 1 November 2020). Temperature and RH were tested every 1 min by an automatic thermohygrograph (RR002, China). Carbon dioxide was measured during three time periods (7:00 to 10:00, 12:00 to 14:00 and 18:00 to 22:00) by a carbon dioxide tester (Testo535, Germany) with a 1 min sampling time. The climate of Xiqing district in Tianjin for the majority of the time during the experiments was obtained from a meteorological forecast and is shown in Table 1.

Nutrient broth agar (NBA, 33 g nutrient AGAR, 1000 mL distilled water, autoclaved at 121 °C for 20 min) and Sabouraud dextrose agar (SDA, 65 g type AGAR, 1000 mL distilled water, 115 °C high-pressure sterilization for 20 min) [37] culture media were used for testing the indoor bacteria and fungi, respectively. Two duplicate plates (90 mm), onto which was poured 15-mL NBA or SDA, were placed at the sampling point at a height of 0.45 m above the floor. The plates were exposed to air for 7 min during a calm period, avoiding periods that involved the making of beds, cleaning of surfaces, start time of activities, etc. Samples were taken in the morning (7:00–10:00), noon (12:00–14:00) and evening (18:00–22:00). The exposure time and the height above the floor were set to ensure that the number of colonies on the plate was in the range 0–100. The number of occupants was recorded at each sampling time. Individual cleaning activities were performed every morning, and a thorough cleaning activity was performed once a week. Next, the petri dishes containing the bacterial samples were placed in an incubator for 48 h at a temperature of 37 °C, while the petri dishes containing fungal samples were placed in an incubator for 120 h (28 °C). In addition, the number of colonies on the plates were counted by eye.

The concentration of airborne bacteria and fungi was calculated by Equation (1) [31,38]. As expressed in Equation (1), the number of microorganisms that naturally settle on a sampling area of 100 cm^2^ in 5 min is equivalent to those contained in 10 L air (A/100 represents the conversion of the sampling area, t/5 represents the conversion of the sampling time and 10/1000 represents the conversion of L to m^3^).

(1) $$C=\frac{N}{\left(\frac{A}{100}\times \frac{t}{5}\times \frac{10}{1000}\right)}=\frac{50000N}{\mathrm{At}}$$

where C is the concentration of airborne microbes, CFU/m^3^; N is the average number of CFUs on the duplicate plates, CFU/dish; A is the sampling area, cm^2^; and t is the exposure time, min.

### 2.3. Dose Rate Assessment

A dose rate assessment model was employed to evaluate the risk of exposure to airborne bacteria for human health by inhalation contact [39]. The dose rate of airborne microbes by inhalation (DRinh, CFU/(kg·d)) can be calculated by Equation (2),

(2) $$\mathrm{DR}=\frac{c\times \mathrm{IR}\times \mathrm{EF}\times \mathrm{ET}}{\mathrm{RW}\times \mathrm{AT}}$$

where c is the microbe concentration in a dormitory room calculated by Equation (1) (CFU/m^3^), IR is inhalation rate (m^3^/d or L/min), EF is exposure frequency (day/annual), ET is exposure time (annual), BW is body weight (kg) and AT is the time expectancy in the room (per day). The parameters for young women were taken from the Exposure Factors Handbook of the Chinese Population (Adult) published by the Ministry of Environmental Protection of China in 2013 [39]. The IR was determined by the intensity of activity during the exposure time and, in this study, it was indexed as 5.1 L/min (“resting”) and 7.6 L/min (“sedentary/passive”), based on the typical activities of students (e.g., sleeping or writing, reading, drawing) [39]. The BW of the adults was determined as 55.6 kg for females. Considering the university terms in China, it was assumed that students stayed on campus for 4 years and studied for 40 weeks per year, usually spending about 11 h in the dormitory on a typical day (8 h for sleeping and 3 h for reading or writing).

### 2.4. Species Identification Method

The technique of 16S ribosomal RNA sequencing (16SrRNA) has been widely used to identify the species of microbes found in indoor air [40,41]. In this study, the bacteria and fungi on the culture dishes sampled in the dormitory were purified. After the sampled culture dish had been cultured for 48 h, the microorganisms in the culture dish were removed with the use of an inoculation ring and coated on the surface of the nutrient broth culture dish or inoculated into a liquid culture medium. The microbe genome was extracted from the liquid culture medium after being cultured for 48 h. The genetic sequencing by 16SrRNA was obtained through comparison with known microbes in the database. Finally, the species of the tested microbes were obtained, and information about the genetic components was verified.

### 2.5. Data Analysis

The average and standard deviation values of bacteria and fungi were obtained by EXECL software. The statistical parameters were calculated by using SPSS and Origin software. The correlations between airborne microbes and the impact factors were analyzed and a statistically significant difference was observed at a confidence level of 95%.

## 3. Results and Discussion

### 3.1. Temperature, RH and CO_2_ of the Tested Female Dorm

**Figure 2:**
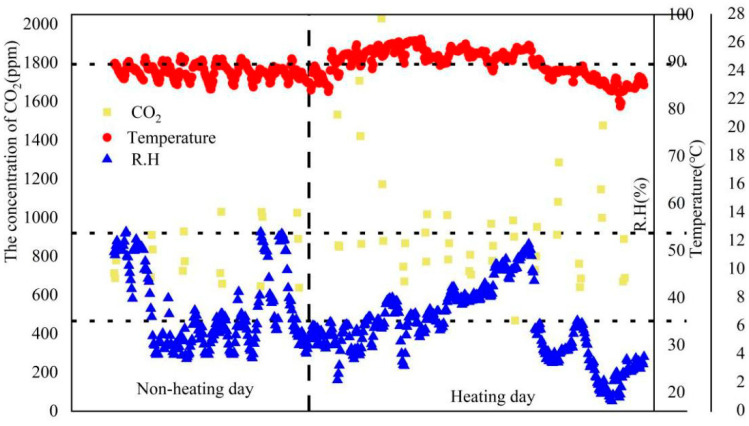
Indoor temperature, R.H. and CO_2_ in the tested dormitory.

The temperature, RH and CO_2_ concentration measured in the selected female dormitory are shown in Figure 2. The maximum temperature exceeded the value (23.0 °C), as recommended in the national standard [36]. The total average values of RH were 38 ± 8.6% and 32.7 ± 7.6% during the unheated and heated periods, respectively. The total average concentrations of CO_2_ were 834 ± 130 ppm (638–1172 ppm, no heating) and 950 ± 291 ppm (469–2030 ppm, heating). However, the mean CO_2_ level with heating in the morning (1061 ppm) was higher than the limit of the national standard (1000 ppm) [36]. Since the frequency of window opening decreased as the outdoor temperature dropped, there was less ventilation at night in the indoor environment; as a result, the maximum concentration occurred in the morning during the heated period.

### 3.2. Total Bacterial and Fungal Counts in the Female Dormitory

#### 3.2.1. Concentration of Microbes Detected during the Sampling Period

**Figure 3:**
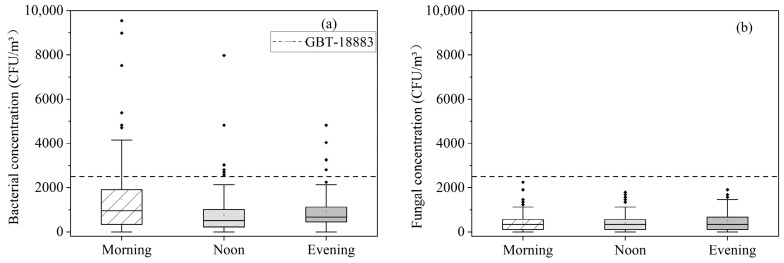
Microbe concentrations in the dormitory in the morning, noon and evening: (**a**) bacteria; (**b**) fungi.

The bacterial and fungi concentrations in the dormitory are shown in Figure 3. The average concentration of bacteria was higher in the morning (930 ± 1681 CFU/m^3^) than at noon (627 ± 991 CFU/m^3^) or in the evening (637 ± 806 CFU/m^3^). The total average concentration of bacteria was 1081 ± 1270 CFU/m^3^. All the mean concentrations during the test times were below the limit of 2500 CFU/m^3^ [36]. Further analysis of the data by a paired *t*-test showed that statistically significant differences were observed in the bacterial concentration comparisons with morning–noon and morning–evening, except noon–evening at a >95% confidence level. This finding resulted from the high occupant density and poor ventilation during sleeping hours [42]. Additionally, the application of makeup by the female occupants may be another reason that the bacterial concentration was the highest in the morning; the influence of makeup application merits further attention in a future study. The average concentrations of fungi in the tested dormitory were 475 CFU/m^3^ (morning), 368 CFU/m^3^ (noon), 467 CFU/m^3^ (evening) and 428 CFU/m^3^ (total). Statistically, the fungal concentrations showed no significant differences during the morning, noon and evening periods by a paired *t*-test at a >95% confidence level. Room occupants and activities lead to more airflow [43], which was conducive to the spread of fungal spores; thus, the concentration of fungi was similar in the morning, noon and evening.

#### 3.2.2. Species of Microbe Tested during the Sampling Period

The four species that appeared the most often on the plates were retrieved from the sampled microbes. For the strains in the tested dormitory, the genetic sequences were obtained by 16SrRNA. The dominant species were Gram-positive microbes. In field studies, *Staphylococcus* and *Micrococcus* appeared to be typical species in indoor air [21,25]. The bacteria found in the sampling were species that are commonly detected indoor air, such as *Staphylococcus hominis* (Gram-positive, the most important in terms of human health effects and the dominant species in universities [44,45]), *Micrococcus yunnanensis* (Gram-positive, found in the human skin microbiome [45]) and *Microbacterium oleivorans* (Gram-positive, identified in human clinical specimens [46]). Meanwhile, the fungi were detected and identified by 16SrRNA and morphology. *Rhodotorula dairenensis*, commonly found in indoor environments [47], was observed in this test.

### 3.3. Effect of Heating on Culturable Airborne Bacterial and Fungal Concentrations

**Figure 4:**
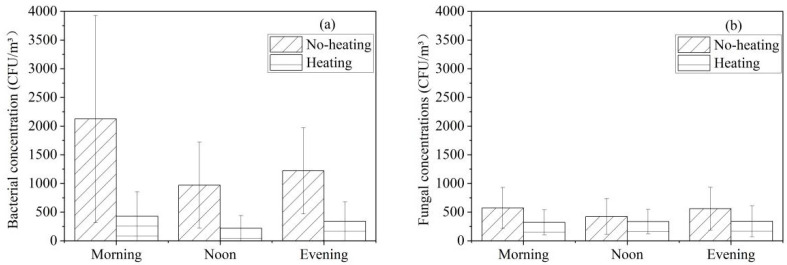
Culturable airborne bacteria and fungi during the unheated and heated periods: (**a**) bacteria; (**b**) fungi.

Figure 4 shows the concentrations of culturable airborne bacteria and fungi during the unheated and heated periods. The bacterial concentration with no heating was higher than that with heating (Figure 4a). The bacterial concentration in the morning (2125 CFU/m^3^ (no heating), 763 CFU/m^3^ (heating)) was the highest during the testing time. The total number and the number of individual species of bacteria collected in the unheated period were found to be greater than those sampled in the heated period. Moreover, at a >95% confidence level, statistically significant differences were obtained for the bacterial concentrations during the morning, noon and evening periods between the unheated and heated times. Based on the sampling method in this study, the increase in activity in the mornings might have caused an increase in particulate material (dust) by re-suspension; the reason being the dust and particles floating in the dormitory due to the stacking of clothes, making of beds and other activities in the morning with full occupancy [19] and the lack of ventilation. Meanwhile, the average concentration of fungi in the morning reached a peak of 574 CFU/m^3^ with no heating, while the highest mean value with heating was 409 CFU/m^3^ at noon (Figure 4b). Since radiator heating reduces moisture in the air, the concentration of fungi with no heating was greater than that with heating [48]. Furthermore, statistically significant differences were observed for the fungal concentrations during the morning period between the unheated and heated times by a *t*-test at a >95% confidence level. This phenomenon might be explained by the fact that there were more activities in the morning than in the noon and evening.

### 3.4. Effect of the Number of Female Occupants on Bacteria and Fungi

**Figure 5:**
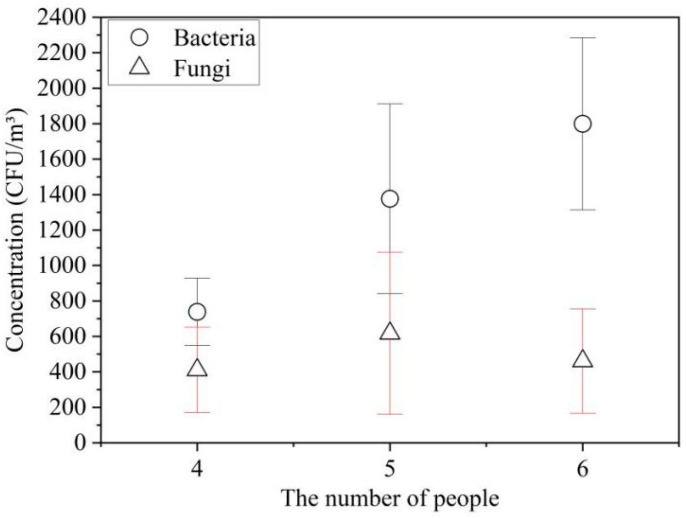
Relationship between the concentrations of microbes and the number of female occupants in the dormitory.

Human occupancy can have an impact on airborne bacteria [49]. Figure 5 shows the relationship between the concentrations of microbes and the number of female occupants in the dormitory. The bacterial concentrations increased with the number of occupants (738 ± 190 CFU/m^3^ (4 occupants), 1377 ± 535 CFU/m^3^ (5 occupants), 1799 ± 485 CFU/m^3^ (6 occupants)) (Figure 5). Further analysis of the data showed that significant differences were observed in all comparisons with the bacterial concentrations of occupants (4–5, 5–6 and 4–6 occupants) at a >95% confidence level. So, in present study, the bacterial concentrations in the dormitory were associated with the number of occupants positively, which has been reported by previous studies that a higher number of occupants resulted in more bacteria in indoor environments [41,49]. In the present study, the fungal concentrations with occupants in the room were 411 ± 241 CFU/m^3^ (4 occupants), 617 ± 457 CFU/m^3^ (5 occupants) and 461 ± 294 CFU/m^3^ (6 occupants). Moreover, at a >95% confidence level, no significant differences were observed in all comparisons with the fungal concentrations and the number of occupants (4–5, 5–6 and 4–6 occupants). No positive correlation was obtained between indoor fungal concentration and the number of occupants in this study. Since there were different numbers of occupants and different factors, this finding was not consistent with previous field results where the concentration of fungi was directly proportional to the number of occupants in the dormitory [50].

### 3.5. Effect of RH on Bacterial and Fungal Concentration

**Figure 6:**
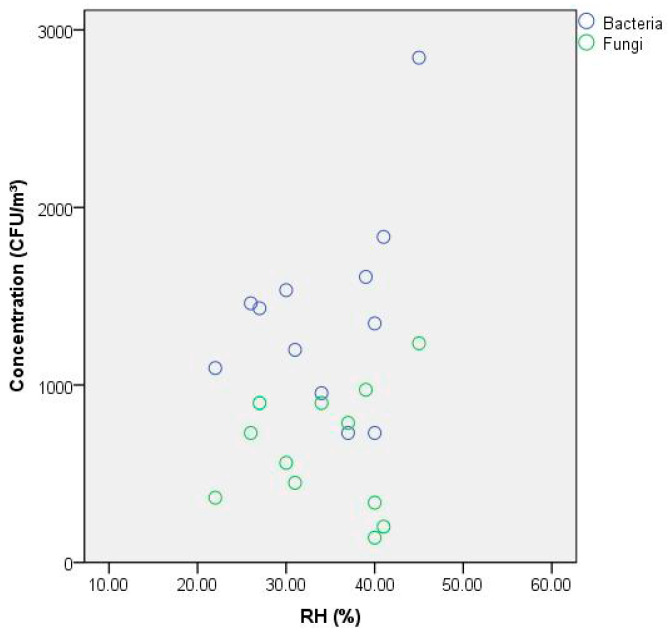
Correlations between the airborne microbe concentration and the RH (6 occupants, sampling time: 21:30–22:30).

Figure 6 displays the correlations between the airborne microbe concentrations and the RH. During further analysis of the data, the bacterial concentrations were correlated with RH (Pearson correlation coefficient: 0.373), statistically. Moreover, no significant differences were observed in bacterial concentrations (*p* = 0.232) at a >95% confidence level. According to the statistical analysis, the correlation between humidity and fungi concentrations was very light (Pearson correlation coefficient: 0.092). In addition, no significant differences were observed in fungal concentrations (*p* = 0.775) at a >95% confidence level. Many field studies have reported that RH is a significant factor that impacts the level of indoor airborne bacteria and fungi. Previous studies have reported that the bacterial and fungal growth was favored with a higher relative humidity [51,52,53]. In the present study, the changes in RH were not as radical among the range of 20–45% when compared to the conditions of other studies. Furthermore, the present study showed that RH had no significant correlation with the airborne bacteria and fungi in the dormitory in our environmental conditions.

### 3.6. Dose Rate of Bacteria and Fungi

The dose rate associated with the inhalation of airborne microbes was assessed during sampling periods, and it was important to understand the exposure risk for the female occupants in the dormitory. Table 2 summarizes the dose rate of airborne bacteria and fungi for different activities during the testing period. In this study, the highest dose rate from exposure to bacteria and fungi was observed during sleeping hours (48 CFU/(Kg·d)), and the dose rate of exposure to bacteria was higher than that of fungi during sleeping and resting periods. Therefore, the concentration of airborne microorganisms to which dormitory occupants were exposed was associated with the activity level in the room. In contrast with gases and particulate matter, there are hundreds of kinds of microorganisms that may be present in the air. The dose rate in this study was aimed only at the total number of active microorganisms.

### 3.7. Limitations

Some limitations were encountered in the present work. One of the restrictions was that the measurement period included only the cooler seasons, autumn and winter, and did not include spring or summer. In addition, few studies have obtained that the peak concentration of the microbes were detected in summer [54] (tested from July to August during the summer holiday, when the students were not staying in the dormitory). Furthermore, because the present study was focused on bacterial and fungal contaminants in less-ventilated spaces under conditions in which occupants spent more time indoors, a cooler sampling period was used for the heated and unheated periods that were evaluated. However, the influence of all four seasons should be explored in future experiments. Another limitation was that a traditional culture-dependent method was selected in this study and some field studies, and cultivation has been frequently employed in the study of bioaerosols. Unfortunately, some small airborne microbes could not be sedimented on the sampling plates, and only a small percentage of the microorganisms (accounting for <1%) in bioaerosols can be cultured. Thus, a potential limitation is that detecting only cultivable microbes led to a gross underestimation of total airborne microorganisms in the field studies.

## 4. Conclusions

In this study, the level, the impact factors (heating, R.H. and occupants) and dose rate of airborne bacteria and fungi were assessed in the female dormitory in university. The average bacterial and fungal concentration in the dormitory satisfied with the national standard limited value, but the species of *Staphylococcus* spp. and *Micrococcus* spp. were detected in the female dormitory. The highest microbe counts were observed in the morning. When the heating was on, the total bacterial and fungal counts were lower than when there was no heating. The heating and number of occupants had an obvious effect on the total bacterial counts. Moreover, RH had correlation with the bacterial concentrations, while no correlation with the airborne fungi, statistically. The dose rate from exposure to airborne microorganisms in the dormitory was associated with the activity level in the dormitory. These results in this paper helped to elucidate the threat of airborne microbe to the health of female occupants and provide guidance for protective measures. 

## Figures and Tables

**Table 1:** Climate of Xiqing district in Tianjin during most of the sampling period.

Sampling Time	Temperature (°C)	Wind Speed (m s^−1^)	RH (%)	Weather
No heating	14 ± 5 °C	3.7 ± 1.5	56 ± 18	Sunny, calm and cloudy
Heating	9 ± 6 °C	4.0 ± 1.9	59 ± 24	Sunny, calm, cloudy, rainy, snowy

**Table 2:** Dose rate from exposure to bacteria and fungi in the female dormitory.

Exposure Pollutants	Dose Rate (CFU/(Kg·d))	
	Sleeping	Reading
Bacteria	48	27
Fungi	19	11

## Data Availability

Not applicable.
